# Endophytic PGPR from Tomato Roots: Isolation, In Vitro Characterization and In Vivo Evaluation of Treated Tomatoes (*Solanum lycopersicum* L.)

**DOI:** 10.3390/microorganisms10040765

**Published:** 2022-04-01

**Authors:** Bastien Cochard, Basile Giroud, Julien Crovadore, Romain Chablais, Lucas Arminjon, François Lefort

**Affiliations:** Plants and Pathogens Group, Research Institute Land Nature and Environment, Geneva School of Engineering, Architecture and Landscape (HEPIA), HES-SO University of Applied Sciences and Arts Western Switzerland, 1254 Jussy, Switzerland; bastien.cochard@hesge.ch (B.C.); basile.giroud@bluewin.ch (B.G.); julien.crovadore@hesge.ch (J.C.); romain.chablais@hesge.ch (R.C.); lucas.arminjon@hesge.ch (L.A.)

**Keywords:** *Bacillus*, biostimulant, endophyte, PGPR, *Pseudomonas*, *Solanum lycopersicum*, sustainable agriculture

## Abstract

Plant-growth-promoting rhizobacteria (PGPR) are soil bacteria colonizing the rhizosphere and the rhizoplane which have an effect on plant growth through multiple chemical compounds. Rhizobacteria with beneficial effects for plants could therefore be used to reduce the dependence on synthetic chemical fertilizers in conventional agriculture. Within this study, 67 endophytic fungi and 49 bacteria were isolated from root samples from 3 different commercial productions: an off-ground tomato production in a greenhouse, an organic production and a conventional production, both in a soil tunnel. Following morphological selection, 12 fungal and 33 bacterial isolates were genetically identified. Thirteen bacterial isolates belonging to nine potential PGPR species were then applied to tomato seedlings established in sterile substrate. The ability of these bacteria to produce indole acetic acid (IAA) and solubilize phosphate was also evaluated. They all were IAA producers and solubilized phosphate. The most interesting strains for growth promotion were found to be the isolates *Pseudomonas palleroniana* B10, *Bacillus subtilis* B25, *Bacillus aryabhattai* B29 and *Pseudomonas fluorescens* B17. The isolates *P. fluorescens* B17, *B. aryabhattai* B29, *B. subtilis* B18 and *Pseudomonas moraviensis* B6 also increased root growth. This study proposed a quick protocol for isolating and testing potential endophytic PGPR that should be characterized further for the direct and indirect mechanisms of growth promotion.

## 1. Introduction

Tomatoes (*Solanum lycopersicum* Linné, 1753) are an important crop worldwide, with an increasing production level on an annual basis [1]. Indeed, its culture, whether soil or soilless, is still predominantly conventional and dependent on synthetic fertilizers and chemical pesticides [2,3,4]. This leads to the biological depletion of soils, groundwater pollution and the development of resistance in pathogens and pests. In a move towards a more sustainable agriculture, using microorganisms, which have a direct beneficial effect on plant growth, is now considered better. Moreover, these microorganisms may indirectly protect crops against pathogens. Integrating these microorganisms into agriculture could help to reduce production costs, increase earliness and increase the share of marketable vegetables [5]. A wide range of plant-growth-promoting rhizobacteria (PGPR) are known to be associated with the rhizosphere of tomatoes and belong to the following genera: *Pseudomonas*, *Bacillus*, *Acinetobacter*, *Streptomyces*, *Micrococcus*, *Azotobacter*, *Flavobacterium* or *Streptococcus* [6,7]. Many studies have shown an increase in the vigor or productivity of different plant species following PGPR application, under normal conditions as well as under stress [8,9]. PGPR have an increasing interest as plant biostimulants in the coming New Green Revolution [10,11]. Intensive and multiple interactions occur in the rhizosphere between plants, soils (or substrates) and soil microorganisms [3,12]. These interactions can significantly influence plant growth and yields. In the rhizosphere, bacteria are the largest component of microbial diversity [7,13]. Rhizobacteria are specific bacteria that actively invade the roots of plants and colonize them at all stages of plant growth.

Rhizobacteria that promote plant growth can be classified into two categories: extracellular plant-growth-promoting rhizobacteria (ePGPR) and intracellular plant-growth-promoting rhizobacteria (iPGPR). The ePGPR can be found in the rhizosphere, on the rhizoplane or in the intercellular spaces of the root cortex. On the other hand, iPGPR are usually found within specialized nodular structures in root cells. In addition, they can be considered as symbiotic species in comparison with ePGPR [14]. Bacteria of the genus *Arthrobacter*, *Azotobacter*, *Azospirillum*, *Bacillus*, *Caulobacter*, *Chromobacterium*, *Erwinia*, *Flavobacterium*, *Micrococcous*, *Pseudomonas* or *Serratia* belong to ePGPR, whereas iPGPR mainly belong to the Rhizobiaceae family, including the genera *Allorhizobium*, *Bradyrhizobium*, *Mesorhizobium* and endophytic *Rhizobium* or *Frankia* [2,15]. There are many known interactions between plants and PGPR, which result in plant growth increases under a variety of environmental and climatic conditions [16]. Generally, PGPR promote the growth of plants by either directly facilitating plants’ acquisition of nitrogen, phosphorus, potassium or other essential elements, or by modulating the levels of phytohormones. They also may indirectly act as biocontrol agents and reduce the effects of inhibited growth and plant development caused by many other pathogenic microorganisms [2]. Improvement in yields and fruit size would result from the PGPR facilitating plant nutrition through direct and indirect methods.

Direct mechanisms used by rhizobacteria are numerous and include nitrogen fixation, phosphate and potassium solubilization, siderophore production and phytohormones production [15,17,18,19,20]. Though nitrogen is the main component of the air (78%), plants are not able to capture nitrogen (N_2_) and fill their needs with ammonia (NH_3_). Some bacteria, owning the complex enzyme system called nitrogenase, are able to fix the atmospheric nitrogen and make it available to plants. Nitrogen-fixing PGPR may transmit it to plants via two different systems: symbiotic and non-symbiotic. Symbiotic bacteria, such as the PGPR genera *Rhizobium*, *Bradyrhizobium*, *Sinorhizobium*, and *Mesorhizobium*, are mostly symbiotic with legumes. On the other hand, non-symbiotic nitrogen-fixing bacteria belong to the genera *Azotobacter*, *Acetobacter*, *Azospirillum*, *Burkholderia*, *Diazotrophicus*, *Enterobacter*, *Gluconacetobacter*, *Pseudomonas* or cyanobacteria [17].

Phosphate solubilization is another direct mechanism. Phosphorus, the second element in importance after nitrogen, plays an important role in many metabolic pathways, such as photosynthesis, energy transfer, signal transduction or cell respiration. Although phosphorus is present in large quantities in all soil types, it is very predominantly found in a precipitated insoluble form of soil, not available to plants, which may only may use the dihydrogen phosphate ion form (H_2_PO_4_^−^) and the monohydrogen phosphate form (HPO_4_^2−^) [15]. Rhizobacteria may solubilize insoluble phosphate in assimilable phosphate by releasing a variety of compounds, such as anions of organic acids, protons, hydroxyl ions or extracellular enzymes [19]. Phosphate solubilizing PGPR include genera such as *Arthrobacter*, *Bacillus*, *Beijerinckia*, *Burkholderia*, *Enterobacter*, *Erwinia*, *Flavobacterium*, *Microbacterium*, *Pseudomonas*, *Rhizobium*, *Rhodococcus and Serratia*. All of these genera could potentially be used to increase phosphate solubilization and thus increase growth and yields [14,15]. Microorganisms in the rhizosphere may also secrete phytohormones such as auxins, cytokinins, gibberellins or ethylene, which stimulate root development and nutrient and water absorption [15]. Indole acetic acid (IAA) is the most common plant auxin that is also synthesized by PGPR. This external IAA allows for increased cell multiplication and mineral nutrients absorption but also stimulates seed germination, root development and resistance to stress. Phytohormones can change the partitioning patterns of assimilation in plants, thus modifying the root growth, fruiting process or fruit development in production conditions [20].

The indirect mechanisms include the capacity of the rhizobacteria to produce antibiotics, volatile organic compounds (VOCs) and exopolysaccharide efficient against a wide range of other microorganisms [3,15,18,21,22,23,24]. *Pseudomonas* bacteria are also able to emit hydrogen cyanide [15,18]. Rhizobacteria may also secrete lytic enzymes such as chitinases, lipases, phosphatases or proteases. Many bacteria also trigger several pathways in the induced resistance system of the plants [2,15,25].

Existing studies on the bacterial diversity of tomato leaves mostly focused on epiphytic bacteria or total phyllospheric bacteria [26,27]. Recently, Romero et al. (2014) [7] addressed this question through metabarcoding and showed that endophytic bacteria in leaves were different from those in roots. If the exact mechanisms of endophyte colonization still need to be understood [28,29], it nevertheless appears that tomato roots could be a source of PGPR present in endophytes, as recently stressed by Anzalone et al. [30].

The present study therefore aimed to isolate cultivable endophytic PGPR from tomato roots and to identify them genetically. After identification, some of these strains were characterized in vitro for their capacities to solubilize insoluble phosphate and to produce the hormone indole acetic acid. The same isolates were then tested in planta in sterile substrates to assess their effects on the growth of tomato seedlings in greenhouse conditions.

## 2. Materials and Methods

### 2.1. Isolation of Endophytes from Roots

Roots of *Solanum lycopersicum* L. were sampled from three distinct tomato greenhouses in the Geneva area. Two of them were run in conventional agriculture modes: a soilless crop in glasshouses for “Serre des Marais, Veyrier” and a soil-based crop under a plastic tunnel for “Serre Chapuis, Veigy, France”. The third one (Serres Pecorini/Pellet, Troinex) was a soil-based crop under a plastic tunnel run in biological agriculture conditions (according to the Swiss good practices of the label BioSuisse). The disinfection of root samples prior to isolation was carried out as follows: roots were rinsed with sterile demineralized water and then cut into 2 cm long pieces, which were then dipped for disinfection in 200 mL 2.5% NaClO under continuous stirring in erlens. The tested disinfection times were 5, 10, 15, 20 and 25 min. All subsequent steps have been carried out in sterile conditions provided by a laminar flow cabinet (Thermo Fisher Scientific, Geneva, Switzerland) using autoclaved glassware and utensils. Then roots were flushed and rinsed three times for 1, 2 and 5 min, respectively, with 200 mL sterile water. Three root subsamples per plant were then plated in Petri dishes (90 mm diam.) and cultivated at room temperature. The screening for diverse colony morphologies was carried out by cultivation on the following media: Luria-Bertani agar (LBA; Roth, Arlesheim, Switzerland), Potato Glucose agar (PGA; Roth, Arlesheim, Switzerland) and an adapted ATCC medium 965, modified by replacing the smashed tomatoes with 28 g of V8 juice (Campbell Soup Co., Camden, NJ, USA). Microorganism colonies appeared after 72 h at 20 °C. This cultivation step resulted in a total of 67 fungal colonies and 48 bacterial colonies, which were then further isolated in pure cultures. After visual observation of the colonies, 33 distinct bacterial isolates were retained for DNA extraction and genetic identification. Selected bacterial isolates were then all kept in duplicates in LB broth:glycerol (50:50) at −20 °C and −80 °C.

### 2.2. DNA Extraction, PCR Amplification and Sequencing

Genomic DNA was extracted following protocols adapted from Ripoll et al. [31]. Nucleic acid quantification was performed with a Nano-Drop ND-1000 Nanospectrophotometer (Thermo Fisher Scientific, Geneva, Switzerland). PCR amplifications were carried out in a total reaction volume of 50 μL in a Biometra^®^ Thermocycler (Goettingen, Germany), with target DNA used at a final concentration of 1 ng/μL. PCR reactions were performed with Bioline BIOTAQ™ DNA polymerase (Labgene Scientific, Châtel-Saint-Denis, Switzerland). Primers ITS 4 and ITS 5 for fungi [32] and 27F and 1492R for bacteria [33,34]) were purchased from Microsynth (Balgach, Switzerland). Conditions for the amplification of the internal transcribed spacer ITS were: an initial denaturation at 95 °C for 3 min, followed by a cycle of 30 s at 95 °C, 30 s at 56 °C, 15 s at 72 °C, repeated 34 times and terminated in 1 min at 72 °C. For amplification of the 16S rDNA gene, the conditions were an initial denaturation at 95 °C for 3 min, followed by 37 cycles (20 s at 95 °C; 15 s at 57 °C; 15 s at 72 °C) and a final step of 1 min at 72 °C. PCR products were finally purified prior to sequencing with the Wizard^®^ SV Gel and PCR Clean-Up System (Promega, Dübendorf, Switzerland). Sanger DNA sequencing was then performed by Microsynth (Balgach, Switzerland). The resulting sequences were subsequently edited with FinchTV v.1.5.0, PhyDE^®^ and MUSCLE [35] and registered in the Nucleotide database of the National Center for Biotechnology information (NCBI, Bethesda, MD, USA) under the accession numbers MH671830-MH671861 for bacterial isolates and MH673602-MH673613 for fungal isolates. DNA sequences were compared to the sequences of the NCBI nucleotide database using the BLASTn tool [36]. Genetic proximity of these 32 selected isolates with 77 sequences of close species isolated from tomato roots [30] was illustrated with a neighbor-joining tree produced with MEGA X v.11, using the Maximum Likelihood method and General Time Reversible model [37].

### 2.3. Selection of Potentially Interesting PGPR Isolates

Given the available literature on tomato rhizosphere and endophytic bacteria, in regard to the genetic identities of the obtained isolates, thirteen isolates belonging to potential PGPR species were retained for in planta evaluation and biochemical characterization. These choices are explained in Section 3.1 and Section 3.2. Besides that, multiple strains of the same species from the same sampled location and displaying the same 16S sequence were considered as one single strain, and therefore only one strain was used in these cases.

### 2.4. In Planta Tests

For setting up in planta tests, tomato seeds of the variety Montfavet H63-5 F1 (HM Clause, Portes-Lès-Valence, France) were sown at 2 seeds each in the seedling substrate Klassman 2 (Klasmann-Deilmann, Geeste, Germany). The substrate, previously sterilized for 20 min at 121 °C, was dispatched in two multipots plates (PMP; 36 × 54 cm; HerkuPlast Kubern, Ering, Germany) of 24 buckets (9 × 9 × 10 cm). Cultures were then thinned after germination in order to leave one plant per bucket. A total of 48 tomato plants were prepared for each of the 13 duplicated treatment modalities, as well as for the negative and positive controls, yielding 720 plants totally.

Bacteria inoculation occurred once plants had germinated, around 5 days after their sewing. For bacterial application, the 13 selected isolates were first cultured in 200 mL LB broth for 48 h at 28 °C in 250 mL Erlenmeyer flasks sealed with Parafilm^TM^ and an aluminum foil stopper under mild agitation at 125 rpm. The resulting volume of culture was enough to inoculate each plant with 4 mL of a high-concentration bacterial culture, to which 50 mL of demineralized sterile water were added to moisten the soil. The positive control was watered with 50 mL of a commercial tomato fertilizer Biorga (50 g/L organic nitrogen, 50 g/L K_2_O, 6 g/L Mg; Hauert, Grossaffoltern, Switzerland) at the recommended concentration of 0.4%. The negative control only received 4 mL sterile LB broth without bacteria and 50 mL sterile water. All modalities were distributed using a completely randomized block design duplicated in the greenhouse, with 2 repetitions per modality yielding 720 seedlings totally. The culture was maintained for 3 weeks in summer 2018 and was watered every 2–3 days depending on the greenhouse conditions. The average temperatures ranged between 18.8 °C at night and 28 °C during the day from 27 July until 17 August, with a peak at 43.5 °C on 10 August. After 3 weeks, aerial and root parts were collected for measuring fresh and dry weights. Aerial and root parts were then dried for 48 h at 55 °C in a drying oven (Memmert, Büchenbach, Germany).

### 2.5. Statistical Analysis

Statistical analyses were performed with Minitab 18 (Minitab^®^). Due to some atypical values obtained after performing the Anderson–Darling normality tests, it appeared not to be possible to use variance analysis tests (ANOVA). For this reason, we use the nonparametric test of Kruskal–Wallis (KW) instead. The KW results are given in the Tables S1 and S2 for roots and aerial parts, respectively. The conditions were as follows: H_0_: all medians are equal; H_1_: at least one median is different; DOF 14: *p*-value 0.00 for adjusted/unadjusted.

### 2.6. Indole Acetic Acid Production

The production of indole acetic acid was assayed using Salkowski’s reagent according to Matsuda et al. (2018) [38]. Overnight LB cultures of bacterial isolates were used to inoculate triplicates consisting of 20 mL LB broth supplemented with 200 mg/L of tryptophan, conducted in 50 mL Falcon tubes. The tubes were then incubated at 20 °C with mild rotating shaking at 120 rpm. Two measurements were taken daily, over 48 h. At the end of this time, 1.5 mL samples were centrifuged 3 min at 10,000 rpm, and 1 mL of the clear supernatant was added to 1 mL of Salkowski reagent (H_2_SO_4_ (7.9 M); FeCl_3_ (12 g/L)) and incubated for 30 min at room temperature in the dark to allow the reaction to develop. Optical densities (ODs) were then read at 530 nm in a Lambda 2 spectrophotometer (Perkin Elmer, Schwerzenbach, Switzerland) and correlated with IAA production according to Glickmann and Dessaux (1995) [39]. The standard range proposed by Ahmad et al. (2008) [40] was optimized to allow accurate measurements at low concentrations. This new range included the following IAA concentrations: 300, 150, 100, 50, 25, 12.5, 6.25, 3.125 mg/mL.

### 2.7. Phosphate Solubilization

The capacity of the selected isolates to solubilize the phosphate was assayed using a protocol adapted from the method of Nautiyal (1999) [41]. For this purpose, the selected isolates were cultivated in triplicates in 10 mL LB broth, in 14 mL Falcon tubes, for 24 h at 20 °C with mild rotating shaking (125 rpm). Then, 1 mL sample of each culture was added to 10 mL of NBRIP (National Botanical Research Institute’s Phosphate) growth medium, which is rich in tricalcium phosphate Ca_3_(PO_4_)_2_, in 14 mL Falcon tubes and then cultured at 25 °C for 72 h under agitation at 180 rpm. Finally, the tubes were centrifuged at 10,000 rpm for 10 min, and 1.5 mL of the supernatant was pipetted into a spectrophotometer cell (1-cm path length). ODs were measured at 600 nm.

## 3. Results

### 3.1. Isolation, Identification and Selection of Endophytes

**Figure 1:**
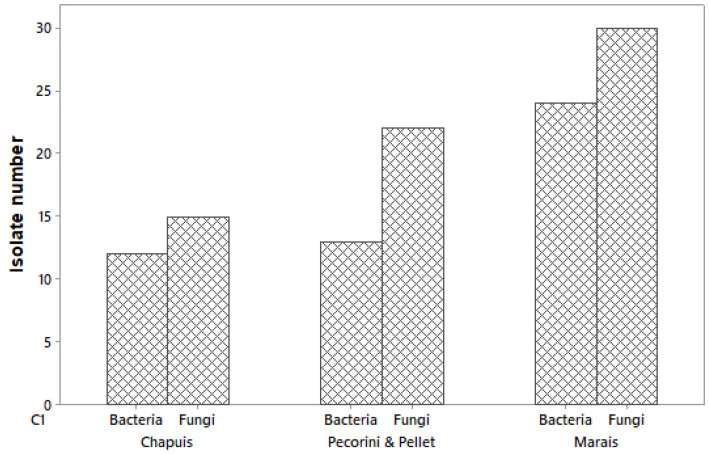
Bacterial and fungal isolates as a function of the samples’ provenances (Serres des Marais, Veyrier, Switzerland; Serres Pecorini and Pellet, Troinex, Switzerland; Serres Chapuis, Veigy, France).

The first round of isolation of the microorganisms yielded 67 total fungal isolates and 49 bacterial isolates, whose distribution is shown in Figure 1. All isolates were observed and compared morphologically, and only distinct isolates were conserved for further DNA extraction and genetic identification. Out of 67 fungal isolates, twelve fungal isolates were chosen for further identification. Six fungal isolates kept from the samples from Serres des Marais revealed to belong to the same species *Plectosphaerella cucumerina*. The six isolates identified from Serres Pecorini and Pellet belonged to the following species: *Fusarium oxysporum*, *Chaetomium elatum*, *Colletotrichum coccodes*, *Acremonium alternatum*, *Plectosphaerella cucumerina* and *Colletotrichum nigrum*. As most of these fungi are potentially pathogenic to tomatoes, they were therefore discarded.

**Figure 2:**
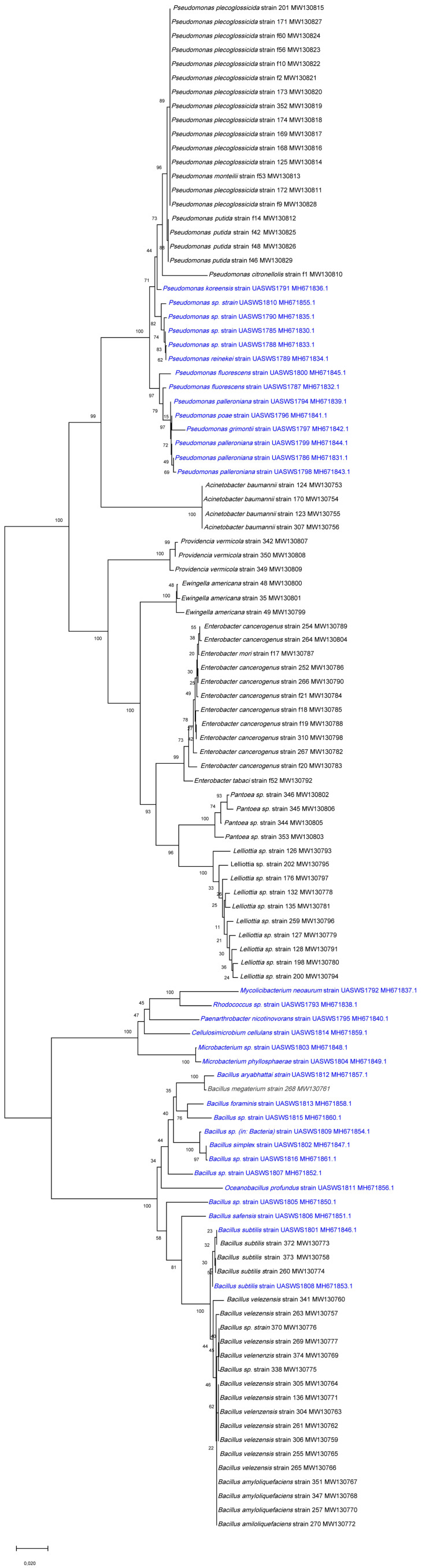
Neighbor-joining tree representing the genetic proximities of the 32 identified bacterial isolates (in blue) to 77 close species and isolates from tomato roots [30]. The tree with the highest log likelihood (−6406.30) is shown. The tree is drawn to scale, with branch lengths measured in the number of substitutions per site.

Out of 49 bacterial isolates, 32 distinct bacterial isolates were conserved and identified (Table 1). Surprisingly, the diversity of endophytic bacteria was also high in off-soil tomato culture. Few species were common between the three tomato culture sources, which would mean that cultivable tomato root endophytes might vary as a function of the tomato variety and the agricultural system. Concerning the bacteria, four isolates from Serres des Marais belonged to *Pseudomonas palleroniana* and were identical in sequence. The two strains of *Pseudomonas reinekei* from Serres des Marais were also genetically identical, as were the two strains of *Microbacterium phylosphaerae* from Serres Pecorini and Pellet. *Pseudomonas fluorescens* was found in tomatoes from Serres des Marais and Serres Pecorini and Pellet, while *Bacillus simplex* and *Bacillus subtilis* were found in Serres Pecorini and Pellet and Serres Chapuis in soil cultures. The diversity of the 32 bacterial isolates is shown in Figure 2, in comparison to 77 endophytic bacterial isolates from tomato roots [30].

### 3.2. Selected Microorganisms for Biochemical and in Planta Tests

Based on the recent literature [5,7,8,13,17,27,30], we retained the following isolates for further biochemical characterization and in planta tests: *Pseudomonas fluorescens* B3, *Pseudomonas moraviensis* B6, *Pseudomonas koreensis* B7, *Rhodococcus degradans* B9, *Pseudomonas palleroniana* B10 (from Serres des Marais, Veyrier), *Pseudomonas fluorescens* B17, *Bacillus subtilis* B18, *Bacillus simplex* B19, *Microbacterium phyllosphaerae* B20, (from Serres Pecorini et Pellet, Troinex), *Bacillus safensis* B23, *Bacillus subtilis* B25, *Bacillus aryabhattai* B29 and *Bacillus simplex* B33 (from Serres Chapuis, Veigy).

More specifically, the selection of these strains was motivated by the available information, which was sometimes scarce for certain species. *Pseudomonas fluorescens* has been long known as a growth promoter in tomato cultures [42,43]. *Pseudomonas moraviensis* has been shown to solubilize phosphate and promote growth in wheat [44]. *Pseudomonas koreensis* is also a phosphate solubilizer and a biocontrol agent of *Pythium ultimum* [45,46]. *Rhodococcus degradans* is little known for promoting plant and fungal growth [47] but belongs to a genus with several species known for degrading synthetic pesticides [48] or promoting plant growth [49]. *Pseudomonas palleroniana* is known for its high capacity to solubilize phosphate [50]. *Bacillus subtilis* was shown to have an effect on tomato seed germination [51], to confer protection against fungal pathogens [29,52], to increase tomato growth [28] and to prevent infestation by the insect *Bemisia tabaci* [53]. *Bacillus subtilis* and *Bacillus simplex* are also PGPR on legumes [54], while *Bacillus simplex* has already been identified as one of the most promising bacterium in tomatoes [55]. The species *Microbacterium phyllosphaerae* has been recently noticed as an endophytic PGPR in hemp [56] and beans [57]. The species *Bacillus safensis*, once isolated from wheat rhizosphere, has been characterized as a very efficient PGPR in corn [58] and a growth promoter in rice in high saline conditions [59], while *Bacillus aryabhattai* (syn. *Priestia aryabhatta*) has been shown to solubilize phosphate, to produce siderophores [60], to be tolerant of oxidative and nitrosative stress and to promote soybean growth by producing phytohormones [61].

### 3.3. In Planta Tests

**Figure 3:**
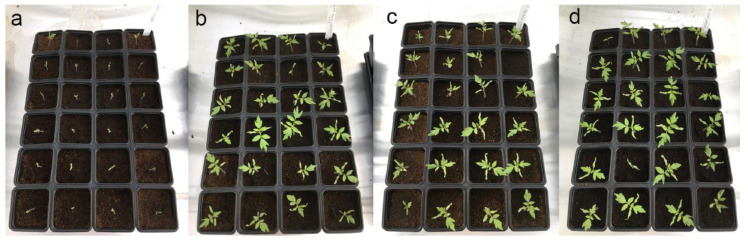
In planta tests on tomato growth promoted by three bacterial strains after 4 days of growth: (**a**) negative control; (**b**) *Bacillus subtilis* B25; (**c**) *Pseudomonas palleroniana* B10; (**d**) *Pseudomonas fluorescens* B17.

**Figure 4:**
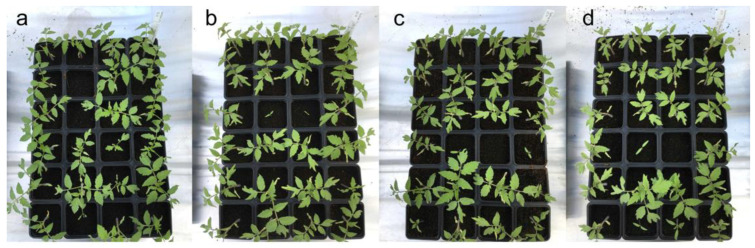
In planta tests on tomato growth promoted by three bacterial strains after 22 days of growth: (**a**) positive control with chemical fertilizer; (**b**) *Bacillus subtilis* B25; (**c**) *Pseudomonas palleroniana* B10; (**d**) *Pseudomonas fluorescens* B17.

**Figure 5:**
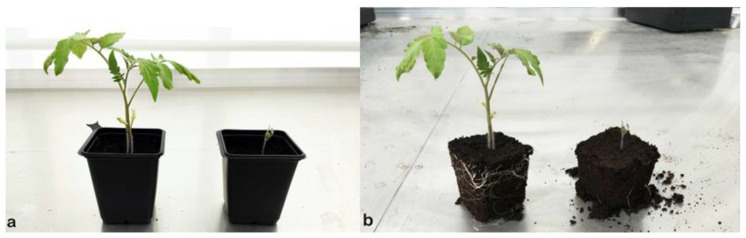
View of potted (**a**) and unpotted (**b**) individual plants. The plant on the left was treated with *Pseudomonas palleroniana* B10. On the right is the negative control (no fertilizer).

Figure 3 and Figure 4 illustrate some of the cultures after 4 days and 22 days of culturing in the greenhouse. Figure 5 shows the details of individual plants (one negative control and one treated plant) and their aerial parts and roots. The treated plant has both more developed aerial parts and roots.

**Figure 6:**
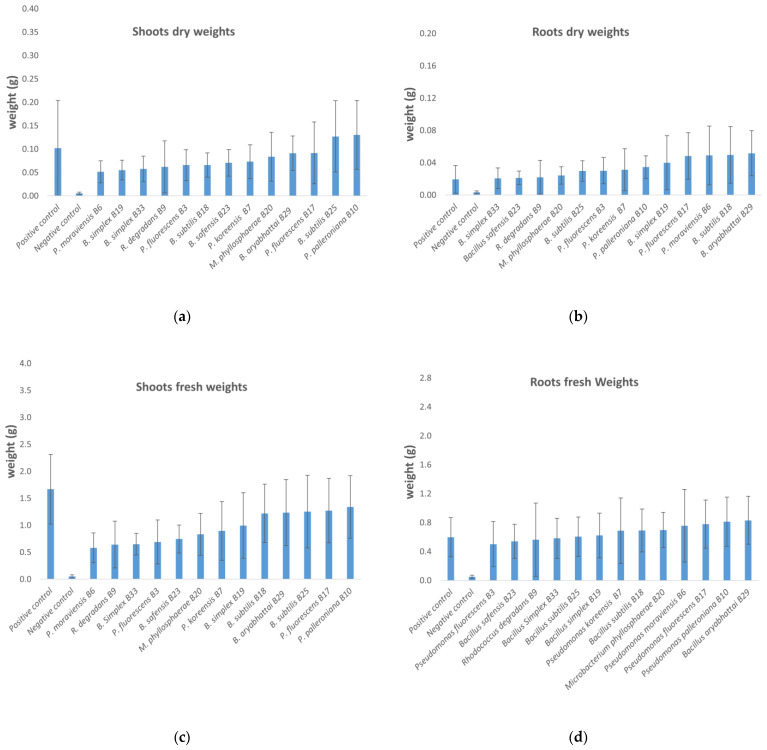
Dry and fresh weights of tomato plants as a function of the treatments: dry weights of (**a**) shoots and (**b**) roots; fresh weights of (**c**) shoots and (**d**) roots. Negative control, T−. Positive control, T+.

The fresh and dry weights of the shoots and roots were assessed for all 13 strains (Figure 6). Weights are given as average weights per plantlet. All isolates produced an increase in the fresh and dry weights of the shoots and roots far higher than the negative controls. Nine of them increased the roots’ fresh weight beyond the positive control (Figure 6d), and seven of the same isolates increased the roots’ dry weight. Concerning the shoots, two isolates *B. subtilis* B25, *P. palleroniana* B10 increased the shoots’ dry weight even beyond the positive control (Figure 6a), while none were better than the positive control for the shoots’ fresh weights (Figure 6c). The data had many atypical values, especially due to the low values of the negative controls, so normality tests (Anderson–Darling) were performed, and the possibly of using ANOVA was withdrawn. The test used is therefore the non-parametric Kruskal–Wallis test, which showed that all the roots’ fresh and dry weights for plants of all treatment types were statistically significantly different than those of the negative control plants. This was also the case for the shoots’ fresh and dry weights, where all isolates displayed a *p*-value less than 1‰ (0.000). For the fresh weights of the root system, the more significantly different effects were obtained with the isolates *B. aryabhattai*, B29, *P. palleroniana* B10 and *P. moraviensis* B6, all having *p*-values less than 1‰. Testing the difference in the roots’ dry weight showed that the isolates *P. fluorescens* B17, *B. aryabhattai* B29 and *B. subtilis* strain B18 produced the most significantly different effects compared to the negative control. For the shoots’ fresh weight, the positive control and then the treatments by the isolates *P. palleroniana* B10, *P. fluorescens* B17 and *B. subtilis* B18 were the most significantly different from the negative control, whereas the isolates *P. palleroniana* B10, *B. subtilis* B25 and *B. aryabhattai* B29 produced the more significant effects on the shoots’ dry weights.

All 13 strains performed far better than the negative control and were equivalent or superior to the positive control. This high difference can be partially explained by phosphate solubilization and the AIA production activities of these strains, but also by the fact that the substrate was sterilized prior to sewing and inoculation of the bacterial isolates. These conditions certainly allowed these bacteria to fully express their positive interactions with the tomato plants without being submitted to the concurrence of the microflora of the substrate. This could also explain the bad performance of the negative control, left without any natural microflora to interact with.

The ranking observed in Figure 6 was confirmed by KW tests for all average weights (Tables S1 and S2), showing that a performant isolate for one variable is not the best isolate for another variable: for instance, the isolate giving the highest average root fresh weight, *P. moraviensis* B6, does not perform the same for the average root dry weight. This is maybe due to the plant retaining more water in its root system under the influence of this bacterium.

### 3.4. Phosphate Solubilization

**Figure 7:**
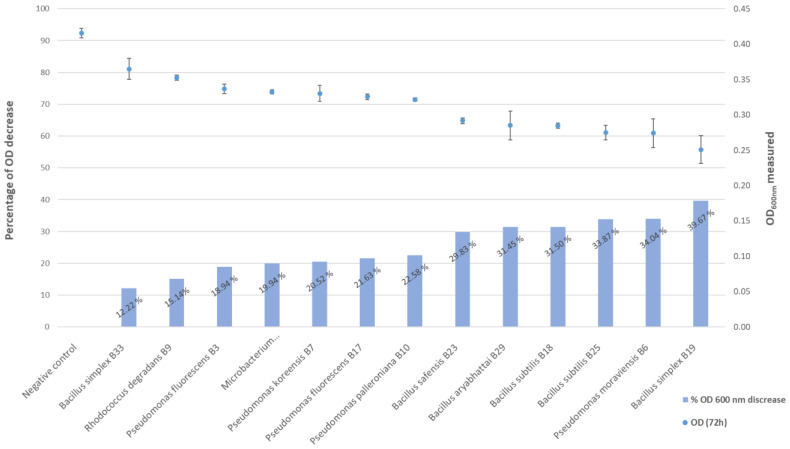
Phosphate solubilization rates given as the OD decrease (%) and OD average of triplicates of the 13 bacterial isolates after a 72-h incubation at 20 °C.

All strains proved able to solubilize phosphate after 72 h of incubation. The quest for phosphate-solubilizing bacteria useful in eco-friendly agriculture is a topical branch [62] and may be useful in tomato culture according to recent studies [63]. In our study, all 13 of the endophytic strains showed the ability to solubilize phosphate (Figure 7), with six isolates, *Bacillus safensis* B23, *Bacillus aryabhattai* B29, *Bacillus subtilis* B18, *Bacillus subtilis* B25, *Pseudomonas moraviensis* B6 and *Bacillus simplex* B19, being very distinctly high phosphate solubilizers. Both *B. subtilis* isolates were very similar in their activity, while the two *B. simplex* isolates behaved very differently, illustrating the need to precisely characterize the phosphate solubilization activity for each retained strain when developing PGPR for agriculture. Experimental data for the percentage decrease in OD_600 nm_ are given in Table S3. We recently showed the correlation of the percentage decrease in OD_600 nm_ with ICP-radial measurements of free phosphate in supernatants [64], which allows this test to be used as a good indicator for the bacterial capacity to solubilize phosphate.

### 3.5. Indole Acetic Acid Production

**Figure 8:**
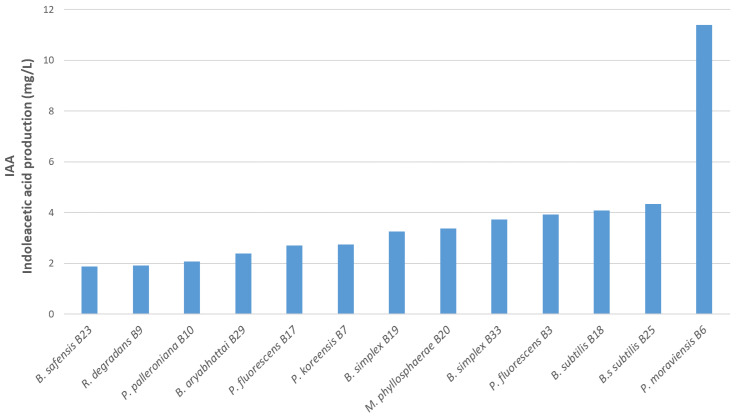
Assay of indole acetic acid production (in mg/L) by the 13 bacterial isolates.

All tested isolates are capable of producing IAA (Figure 8). One isolate, *Pseudomonas moraviensis* B6, exhibited the largest production (11.38 mg/L). The other 12 isolates were similar concerning IAA production. Three of them, *B. subtilis* B18 (4.08 mg/L), B25 (4.34 mg/L) and *P. fluorescens* B3 (3.93 mg/L), showed to be good IAA producers. Concerning IAA production, the two *B. simplex* isolates from two different sources were very similar, although they expressed different capacities to solubilize phosphate. According to Ahmad et al. (2008) [39], the production of IAA is genotype-dependent. Our results showed that, for a tryptophan concentration of 200 mg/L, IAA production is comparable to the results described by Ahmad et al. (2008) [39], i.e., between 3.53 and 6.1 mg/L AIA was produced after 48 h. Experimental data for OD_530 nm_ and its equivalence in IAA (mg/L) as measured at different incubation times are given in Tables S4 and S5.

## 4. Discussion

Growth stimulation results after 22 days are very close to those obtained with the positive control (chemical fertilizer), indicating that the bacteria have a fertilization action equal to the chemical fertilizers. The fact that these trials were run in sterile substrates also indicates that these bacterial strains may therefore help plants to optimize the use of the substrate. This could mean that fewer fertilizers might be used when establishing a culture.

The present study also completes the available knowledge on the bacterial endophytes of tomato roots, confirming many species as endophytes of tomato roots as observed in other works mentioned above, but with cultivable species also not yet observed in tomatoes, such as *Oceanobacillus profundus*, *Mycolicibacterium neoaurum*, *Pseudomonas moraviensis*, *Pseudomonas koreensis*, *Rhodococcus degradans*, *Paenarthrobacter nicotinovorans*, *Pseudomonas poae*, *Pseudomonas grimontii*, *Microbacterium phyllosphaerae*, *Bacillus wiedmannii* and *Bacillus subterraneus.*

The diversity of such cultivable PGPR also seems to vary as a function of the tomato variety and the agricultural system, which was applied in this study. Lemanceau et al. [65] observed that some plants were able to attract endophytic microorganisms that could be favorable to them. They demonstrated that flax (*Linum usitatissimum*) and tomato were able to attract specific strains of *Pseudomonas*, but this was low in abundance and only in non-rhizosphere soil. The selective recruitment of PGPR by plants would certainly be multifactorial and depend on the culture conditions and the availability of particular species in the proximity of the plants. A recent study by Friman et al. [66] showed, for example, that plants recruit certain rhizobacteria in response to an insect attack on its aerial parts, in order to increase its natural defenses against the aggressor. If much research has been conducted to demonstrate the potential growth-promoting or other beneficial effects of endophytic bacteria for tomatoes, however, studies determining which endophytic bacteria have the best potential for growth promotion or biocontrol agents have been rare. In a similar work, Tian et al. [67] identified 49 strains of different endophytic bacteria species from the tomato root microbiome, all belonging to the phylla of firmicutes and proteobacteria. Proteobacteria orders included Pseudomonadales, Enterobacteriales, Rhizobiales, Burkholderiales and Xanthomonadales. A total of 31 of the 49 strains demonstrated antagonistic activity against microbial targets, and most of the endophytes with antimicrobial activity were *Bacillus* and *Pseudomonas* strains. Most isolates also had a capacity to promote growth, but only three strains produced AIA when grown on media containing L-tryptophan. Seventeen strains were nitrogen-fixing, with the *Bacillus* and *Rhizobium* species being the main represented species. These results showed that most of the beneficial endophytic root bacteria tested belonged to three main genera, *Pseudomonas*, *Bacillus* and *Rhizobium* [67], while we did not isolate any *Rhizobium* strains in the present study. More recently, it was shown that the nature of the sampling site could also play an important role in modulating the bacterial diversity of the tomato rhizosphere [68]. This could explain why the rare studies on the diversity of cultivable endophytic bacteria [30,65,67,69] isolated from tomato roots show different spectra of bacteria species.

The diversity we observed in our experiments leads us to think that these different species may interact between themselves and the plants in order to favorize plant growth. From the available literature, it seems that the *Bacillus* and *Pseudomonas* species could be more efficient biofertilizers when used together [8,70,71,72]

## 5. Conclusions

Future works should therefore focus on finding the optimal combinations of the isolates characterized in this study, as well as prospecting the combinations of bacteria with fungi, such as was conducted for the *B. amyloliquefaciens* and *Trichoderma* species [72]. It will be highly rewarding to use these bacteria due to their effect on growth promotion, but characterization of their antifungal activities should also be pursued since it appears more and more that some species can harbor these two assets [64,66].

The diversity of microbes found in *Solanum lycopersicum* L. has been approached on some occasions [6,8,30,67,69,73], and some bacterial biofertilizers are already available on the market. Out of the thirteen endophytic isolates from tomato roots studied, three of them, *B. subtilis* B25, *P. palleroniana* and *P. fluorescens* B17, are highly promising and should now be developed further to find their place on the global biostimulants markets. Such bacteria could also enrich our knowledge of bacterial root endophytes linked to plant growth promotion [74,75], which is determinant for developing a new agriculture able to face an increasing number of problems such as recurrent droughts and phosphate shortages.

## Figures and Tables

**Table 1:** Identities of bacterial isolates from tomato roots, codes, NCBI GenBank accessions, UASWS codes and provenances (1. Serres des Marais, Veyrier, Switzerland; 2. Serres Pecorini and Pellet, Troinex, Switzerland; 3. Serres Chapuis, Veigy, France). UASWS = University of Applied Sciences and Arts Western Switzerland.

Genus	Specie	Isolate Code	NCBI GenBank Accession	UASWS Code	Provenance
*Pseudomonas*	*reinekei*	B1	MH671830.1	UASWS1785	1
*Pseudomonas*	*palleroniana*	B2	MH671831.1	UASWS1786	1
*Pseudomonas*	*fluorescens*	B3	MH671832.1	UASWS1787	1
*Pseudomonas*	*reinekei*	B4	MH671833.1	UASWS1788	1
*Pseudomonas*	*reinekei*	B5	MH671834.1	UASWS1789	1
*Pseudomonas*	*moraviensis*	B6	MH671835.1	UASWS1790	1
*Pseudomonas*	*koreensis*	B7	MH671836.1	UASWS1791	1
*Mycolicibacterium*	*neoaurum*	B8	MH671837.1	UASWS1792	1
*Rhodococcus*	*degradans*	B9	MH671838.1	UASWS1793	1
*Pseudomonas*	*palleroniana*	B10	MH671839.1	UASWS1794	1
*Paenarthrobacter*	*nicotinovorans*	B11	MH671840.1	UASWS1795	1
*Pseudomonas*	*poae*	B12	MH671841.1	UASWS1796	1
*Pseudomonas*	*grimontii*	B13	MH671842.1	UASWS1797	1
*Pseudomonas*	*palleroniana*	B15	MH671843.1	UASWS1798	1
*Pseudomonas*	*palleroniana*	B16	MH671844.1	UASWS1799	1
*Pseudomonas*	*fluorescens*	B17	MH671845.1	UASWS1800	2
*Bacillus*	*subtilis*	B18	MH671846.1	UASWS1801	2
*Bacillus*	*simplex*	B19	MH671847.1	UASWS1802	2
*Microbacterium*	*phyllosphaerae*	B20	MH671848.1	UASWS1803	2
*Microbacterium*	*phyllosphaerae*	B21	MH671849.1	UASWS1804	2
*Bacillus*	*wiedmannii*	B22	MH671850.1	UASWS1805	2
*Bacillus*	*safensis*	B23	MH671851.1	UASWS1806	3
*Bacillus*	*subterraneus*	B24	MH671852.1	UASWS1807	3
*Bacillus*	*subtilis*	B25	MH671853.1	UASWS1808	3
*Bacillus*	*simplex*	B26	MH671854.1	UASWS1809	3
*Pseudomonas*	sp.	B27	MH671855.1	UASWS1810	3
*Oceanobacillus*	*profundus*	B28	MH671856.1	UASWS1811	3
*Bacillus*	*aryabhattai*	B29	MH671857.1	UASWS1812	3
*Bacillus*	*foraminis*	B30	MH671859.1	UASWS1813	3
*Cellulosimicrobium*	*cellulans*	B31	MH671859.1	UASWS1814	3
*Bacillus*	*solani*	B32	MH671860.1	UASWS1815	3
*Bacillus*	*simplex*	B33	MH671861.1	UASWS1816	3

## Data Availability

DNA sequences of the organisms isolated in this work have been registered in the Nucleotide database of the National Center for Biotechnology information (NCBI) under the accession numbers MH671830 to MH671861 for bacterial isolates and MH673602 to MH673613 for fungal isolates.

## Supplementary Materials

The following supporting information can be downloaded at: https://www.mdpi.com/article/10.3390/microorganisms10040765/s1, Table S1: KW test results for root dry and fresh weights; Table S2: KW test results for aerial parts’ dry and fresh weights; Table S3: IAA production—DO_530 nm_ measured at different incubation times; Table S4: IAA production—Equivalence in IAA (mg/L) measured at different incubation times; Table S5: Phosphate solubilization—OD_600 nm_ decrease (%).

## Computer Code and Software

FinchTV v.1.5.0 software (Geospiza, Inc., Seattle, WA, USA) is available at https://digitalworldbiology.com/FinchTV, accessed on 18 February 2022. PhyDE^®^ software is available at http://www.phyde.de/, accessed on 18 February 2022). MEGA X v.11. is available at https://www.megasoftware.net/, accessed on 18 February 2022.
